# Spatiotemporal analysis of tumour-infiltrating immune cells in biliary carcinogenesis

**DOI:** 10.1038/s41416-022-01933-0

**Published:** 2022-09-06

**Authors:** Alphonse Charbel, Luca Tavernar, Thomas Albrecht, Fritz Brinkmann, Joanne Verheij, Eva Roos, Monika Nadja Vogel, Bruno Köhler, Christoph Springfeld, Alexander Brobeil, Peter Schirmacher, Stephan Singer, Arianeb Mehrabi, Stephanie Roessler, Benjamin Goeppert

**Affiliations:** 1 Institute of Pathology, University Hospital Heidelberg, Heidelberg, Germany; 2 Liver Cancer Centre Heidelberg (LCCH), Heidelberg, Germany; 3 Department of Pathology, Amsterdam UMC, University of Amsterdam, Amsterdam, The Netherlands; 4 Diagnostic and Interventional Radiology, Thoraxklinik at University Hospital Heidelberg, Heidelberg, Germany; 5 Department of Medical Oncology, National Centre for Tumour Diseases, University Hospital Heidelberg, Heidelberg, Germany; 6 Tumor Bank Unit, Tissue Bank of the National Center for Tumor Diseases, Heidelberg, Germany; 7 Institute of Pathology, University of Tübingen, Tübingen, Germany; 8 Department of General, Visceral and Transplantation Surgery, University Hospital Heidelberg, Heidelberg, Germany; 9 Institute of Pathology and Neuropathology, Hospital RKH Kliniken Ludwigsburg, Ludwigsburg, Germany

**Keywords:** Cancer microenvironment, Immunoediting, Biliary tract cancer

## Abstract

**Background:**

Intraductal papillary neoplasms (IPN) and biliary epithelial neoplasia (BilIN) are well‐defined precursor lesions of biliary tract carcinoma (BTC). The aim of this study was to provide a comprehensive characterisation of the inflammatory microenvironment in BTC precursor lesions.

**Methods:**

Immunohistochemistry was employed to assess tumour-infiltrating immune cells in tissue samples from patients, for whom precursor lesions were identified alongside invasive BTC. The spatiotemporal evolution of the immune microenvironment during IPN-associated carcinogenesis was comprehensively analysed using triplet sample sets of non-neoplastic epithelium, precursor lesion and invasive BTC. Immune-cell dynamics during IPN- and BilIN-associated carcinogenesis were subsequently compared.

**Results:**

Stromal CD3^+^ (*P* = 0.002), CD4^+^ (*P* = 0.007) and CD8^+^ (*P* < 0.001) T cells, CD20^+^ B cells (*P* = 0.008), MUM1^+^ plasma cells (*P* = 0.012) and CD163^+^ M2-like macrophages (*P* = 0.008) significantly decreased in IPN compared to non-tumorous biliary epithelium. Upon transition from IPN to invasive BTC, stromal CD68^+^ (*P* = 0.001) and CD163^+^ (*P* < 0.001) macrophages significantly increased. In contrast, BilIN-driven carcinogenesis was characterised by significant reduction of intraepithelial CD8^+^ T-lymphocytic infiltration from non-tumorous epithelium via BilIN (*P* = 0.008) to BTC (*P* = 0.004).

**Conclusion:**

IPN and BilIN are immunologically distinct entities that undergo different immune-cell variations during biliary carcinogenesis. Intraepithelial CD8^+^ T-lymphocytic infiltration of biliary tissue decreased already at the IPN-precursor stage, whereas BilIN-associated carcinogenesis showed a slowly progressing reduction towards invasive carcinoma.

## Background

Cholangiocarcinoma (CCA) and gallbladder adenocarcinoma (GBC) constitute a biologically heterogeneous group of malignancies, collectively acknowledged as biliary tract cancer (BTC). CCA is further categorised into intrahepatic, perihilar and distal subtypes according to the anatomical location of the tumour in the biliary tree [1, 2]. Moreover, it is nowadays broadly acknowledged that each BTC subtype, in particular extrahepatic and intrahepatic ones, harbour unique clinical and distinct molecular features [3–5]. Particularly for BTC subtypes arising from precursor lesions, therapeutic options are limited, and diagnosis is often made at an advanced, unresectable stage. The scarcity of effective systemic therapy regimens, coupled with a high incidence of recurrence after resection, has contributed to poor 5-year survival rates of less than 10% [6, 7]. A hallmark of BTC carcinogenesis is the association with various chronic inflammatory diseases such as primary sclerosing cholangitis (PSC), ulcerative colitis, chronic hepatitis B or C virus infections, liver fluke infection, hepatolithiasis, liver cirrhosis and chronic cholecystitis [8, 9]. Despite recent efforts to characterise the molecular spectrum of biliary tract malignancies and the emergence of targeted therapies for advanced CCA harbouring specific alterations [10–12], the survival benefit remains modest. A comprehensive understanding of BTC pathogenesis is therefore needed for guiding new therapeutic modalities [3, 13].

The inflammatory tumour microenvironment (TME) is increasingly recognised as an epicentre of cancer dynamics. Tumour initiation, growth and dissemination are regulated by complex interactions between various effectors of the innate and adaptive immune system [14, 15]. Inflammation is growingly considered as a principal modulator of tumour evolution, driving the transition of cancer-neighbouring stromal cells from a dormant to a pro-tumorigenic state [16, 17]. Increased cell plasticity within the TME moulds immune-cell activation states and potentiates antitumour immunity [18, 19]. However, the spatiotemporal features of the crosstalk between tumour and immune cells during the progression of carcinogenesis have not been completely elucidated, and comprehensive analyses of the TME at precancerous states are scarce. Therefore, we aimed to characterise the spatiotemporal evolution of tumour-infiltrating immune cells quantitatively and qualitatively during the early steps of biliary carcinogenesis.

Premalignant, non-invasive lesions of the biliary tract are gaining interest as recent attempts to understand the multistep model of biliary carcinogenesis emerge [20–22]. The two main types are the frequently observed, microscopically visible biliary intraepithelial neoplasm (BilIN) and the less common, macroscopically visible intraductal papillary neoplasms (IPN). IPN comprise the intraductal papillary neoplasm of the bile duct (IPNB) and the grossly similar but morphomolecularly distinct subtype of intraductal tubulopapillary neoplasm of the bile duct (ITPN) [23, 24]. These lesions are classified according to the degree of cytoarchitectural atypia in low- and high-grade neoplasia. Furthermore, the lining epithelium of the papillary neoplasms is further subclassified into the pancreatobiliary, intestinal, gastric or oncocytic type [25]. In a recent multi-omics approach, we demonstrated that IPNB and ITPN are early forms of biliary carcinogenesis, display common and distinct mutation profiles, and are associated with favourable patient outcome [22]. In addition, we previously characterised the immune infiltrates in non-IPNB/ITPN BTC, thereby revealing a significantly longer overall survival in patients with high intraepithelial CD4^+^, CD8^+^ or FOXP3^+^ T lymphocytes densities, particularly in extrahepatic CCA and GBC but not in intrahepatic CCA [26, 27]. Given the currently evolving histomorphological and molecular understanding of BTC precursor lesions [4, 28] and the recent development of immunotherapeutic strategies against BTC [29, 30], we aimed to investigate the spatiotemporal distribution of tumour-infiltrating immune cells during biliary multistep tumorigenesis.

## Methods

### Patient selection

This study comprised 139 patients with high-grade biliary tract cancer precursor lesions, of which 65 cases had intraductal papillary neoplasms of the biliary tract (IPN) and 74 biliary intraepithelial neoplasia (BilIN). All patients underwent bile duct and/or liver surgery at the University Hospital Heidelberg between 1996 and 2020. Patients who presented with active synchronous malignancies at the time of diagnosis, had received radiochemotherapy before surgery, were formerly diagnosed with primary sclerosing cholangitis or had a history of biliary stent implantation were not included. Overall survival data were available for 96 patients. IPNB and BilIN were diagnosed and graded according to the current World Health Organization (WHO) criteria [23], and ITPN of the bile duct was diagnosed and classified using the criteria of AFIP [24]. Pathological tumour-node-metastases (TNM) staging of associated invasive BTC tissue was reassessed according to the 8th edition of Union for International Cancer Control (UICC) [1]. Ethical approval was received by the institutional ethics committee of Heidelberg University (S-519/2019).

### Tissue selection, tissue microarray construction and immunohistochemical staining

**Fig. 1:**
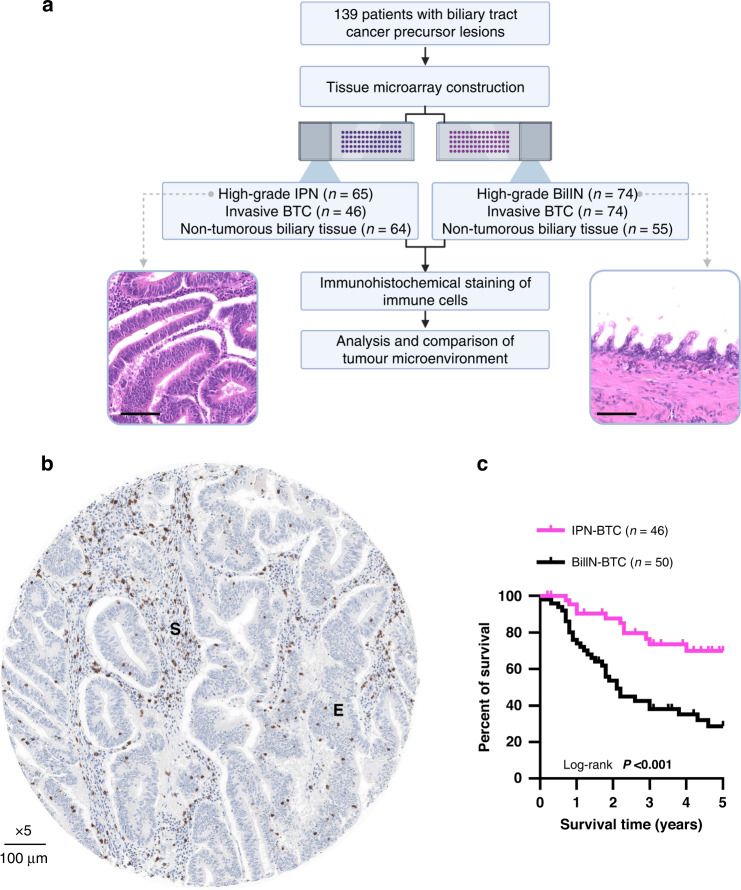
Study design and cohort characterisation. **a** A total of 139 patients with biliary tract cancer precursor lesions were included in this study: *n* = 65 high-grade intraductal papillary neoplasms of the biliary tract (IPNB/ITPN), *n* = 74 high-grade BilIN and their associated invasive and non-tumorous counterparts were selected for tissue microarray construction. Scale bars represent 100 µm. Images were acquired at ×20 magnification. **b** Representative immunohistochemical staining of a tissue microarray dot showing CD8^+^ lymphocytes in the stromal (S) and intraepithelial (E) compartments of IPNB tissue. **c** Kaplan–Meier survival curves of IPN- and BilIN-associated cases.

Formalin-fixed paraffin-embedded (FFPE) tissue specimens from the selected patients were provided by the Tissue Bank of the National Centre for Tumour Diseases (NCT, Heidelberg, Germany). In total, intraindividual triplet patient samples of 65 IPN (54 IPNB, 11 ITPN), 46 associated invasive BTC, and 64 corresponding non-tumorous biliary tissue were included. Only 6 IPNB samples were not associated with an invasive BTC and were therefore labelled as UICC 0 (Table 1). Similarly, 74 BilIN, their 74 associated invasive BTC and 55 corresponding non-tumorous biliary tissue samples were selected from our previous study as a comparison group ([26], Fig. 1a).

**Table 1:** Clinicopathological characteristics of patients with high-grade intraductal papillary neoplasms of the biliary tract (IPN) or high-grade biliary intraepithelial neoplasia (BilIN).

	Total	IPN	BilIN	*P* value
Parameter, *N* (%)	139 (100.0)	65 (46.8)	74 (53.2)	
Age				
Median, years [IQR]	68 (59–74)	64 (54–73)	69 (62–75)	**0.042**^†^
Sex				
Male	82 (59.0)	40 (61.5)	42 (56.8)	
Female	57 (41.0)	25 (38.5)	32 (43.2)	0.607^‡^
Localisation				
Intrahepatic	23 (16.5)	20 (30.8)	3 (4.1)	
Perihilar	34 (24.5)	17 (26.2)	17 (23.0)	
Distal	50 (36.0)	28 (43.1)	22 (29.7)	
Gallbladder	32 (23.0)	0 (0.0)	32 (43.2)	**<0.001**^§^
UICC^¶^				
UICC 0	6 (4.3)	6 (9.2)	0 (0.0)	
UICC 1	15 (10.8)	13 (20.0)	2 (2.7)	
UICC 2	45 (32.4)	22 (33.8)	23 (31.1)	
UICC 3	33 (23.7)	9 (13.8)	24 (32.4)	
UICC 4	13 (9.4)	4 (6.2)	9 (12.2)	
NA	27 (19.4)	11 (16.9)	16 (21.6)	**<0.001**^§^
pT				
Tis	6 (4.3)	6 (9.2)	0 (0.0)	
T1	26 (18.7)	21 (32.3)	5 (6.8)	
T2	67 (48.2)	29 (44.6)	38 (51.4)	
T3	32 (23.0)	7 (10.8)	25 (33.8)	
T4	8 (5.8)	2 (3.1)	6 (8.1)	**<0.001**^§^
pN				
N0	55 (39.6)	38 (58.5)	17 (23.0)	
N1	53 (38.1)	13 (20.0)	40 (54.1)	
N2	1 (0.7)	1 (1.5)	0 (0.0)	
NA	30 (21.6)	13 (20.0)	17 (23.0)	**<0.001**^§^
M				
M0	128 (92.1)	61 (93.8)	67 (90.5)	
M1	11 (7.9)	4 (6.2)	7 (9.5)	0.542^‡^
G				
G1	4 (2.9)	2 (3.1)	2 (2.7)	
G2	103 (74.1)	46 (70.8)	57 (77.0)	
G3	26 (18.7)	11 (16.9)	15 (20.3)	
NA (Tis)	6 (4.3)	6 (9.2)	0 (0.0)	0.065^§^
Invasive component				
Yes	133 (95.7)	59 (90.8)	74 (100.0)	
No	6 (4.3)	6 (9.2)	0 (0.0)	**0.009**^‡^

*IQR* interquartile range. Values in bold indicate statistical significance.

^†^Mann–Whitney *U* test; ^‡^Fisher’s exact test; ^§^χ² test.

^¶^8th edition; cases with pNx had no lymph nodes resected, therefore, UICC status could not be assessed.

Immunohistochemical staining for subsequent analysis was conducted after tissue microarray (TMA) fabrication. Two regionally distinct tissue cores (each 1 mm in diameter) of each defined and marked regions were punched out from the donor blocks and embedded into a new paraffin array block using an automated tissue microarrayer (TMA Grand Master Fa. Sysmex, Germany). For the TMA fabrication of precursor lesions and invasive carcinoma blocks, two independent representative regions were sampled. For immunohistochemistry (IHC), 3-μm sections of the TMA were cut, deparaffinized and rehydrated. For heat-induced epitope retrieval Ultra CC1 (Cell Conditioning Solution, Ventana Medical Systems, Tucson, AZ, USA) was used. After blocking of endogenous peroxidase, slides were incubated with the following primary antibodies: anti-CD3 (clone 2GV6), anti-CD4 (clone SP35), anti-CD20 (clone L26), anti-CD163 (clone MRQ-26), anti-CD56 (clone MRQ-42), anti-MUC2 (clone 760-4388), anti-CDX2 (clone EPR2764Y), anti-synaptophysin (clone MRQ-40) and anti-chromogranin A (clone LK2H10) from Ventana Medical Systems (Tucson, AZ, USA); anti-CD8 (clone C8/144B), anti-MUM1 (clone MUM1-P) and anti-CD68 (PG-M1) from DAKO (Glostrup, Denmark); anti-MUC5AC (clone CLH2) and anti-MUC6 (clone CLH5) from Santa Cruz Biotechnology (Dallas, TX, USA); and anti-MUC1/EMA (clone M0613) from Agilent Dako (Santa Clara, CA, USA). Biotin-free OptiView DAB IHC Detection Kit (Ventana Medical Systems, Tucson, AZ, USA), including OptiView Universal Linker, OptiView HRP Multimer and DAB-Chromogen was used. Finally, the slides were counterstained with haematoxylin.

### Tissue microarray evaluation and immune-cell quantification

The histomorphological identification, grading and subclassification of biliary tract precursor lesions were supported by immunohistochemical analyses of MUC1, MUC2, MUC5AC, MUC6 and CDX2 and were conducted by two specialised pathologists (BG, LT) who were blinded to patients’ clinicopathological data [23]. All ITPN showed a predominant tubulopapillary or trabecular architecture and were MUC5AC negative by immunohistochemical analysis. In addition, neuroendocrine neoplasia was ruled out by performing immunohistochemistry of synaptophysin and chromogranin A.

Stained TMA slides were digitalised using the Aperio AT2 DX System (Leica Biosystems, Vista, CA, USA). Histopathological quantification of immune-cell markers was then performed manually and semi-automatedly using QuPath software version 2.0.3 [31], hereby lowering interobserver discrepancy and achieving higher robustness of analysis. The immuno-staining was in all instances evaluated in conjunction with cell morphology to avoid misidentification. CD3^+^, CD4^+^, CD8^+^ T lymphocytes, CD20^+^ B lymphocytes, MUM1^+^ plasma cells, CD68^+^ macrophages, CD163^+^ M2-like macrophages and CD56^+^ natural killer (NK) cells were analysed in the IPN cohort. Data on immune-cell distribution in BilIN-associated carcinogenesis was retrieved from our previous study [26]. Here, the quantification of CD4^+^, CD8^+^, CD20^+^ and CD68^+^ cells from cases with simultaneously available non-tumorous, BilIN and invasive BTC tissue were available only. Total cell counts of tissue-infiltrating immunohistochemically positive cells were quantified in both cohorts as a numerical count per TMA dot. The proportion of intraepithelial immune cells within a representative area containing 100 biliary epithelial cells was similarly evaluated in both groups. A numerical quantification of the stromal compartment was further included for the IPN group. Immune cells observed in vessels and lymphoid follicles (aggregates) were excluded from counting. For technical reasons, some TMA dots could not be evaluated with the necessary precision. Therefore, the number of cases included varied between the statistical analyses of the different immune-cell types. A representative TMA dot picture of stromal and intraepithelial CD8^+^ lymphocytes is shown in Fig. 1b. In addition, immune-cell quantification was performed on whole-slide sections of arbitrarily selected cases for validation purposes (Supplementary Fig. S1).

### Statistical analysis

Statistical analysis was performed using GraphPad Prism version 9.3.0 for Windows (GraphPad Software, San Diego, CA, USA) and R (https://www.r-project.org/; version 4.1.1) [32]. Differences between continuous variables were assessed for significance by the Kruskal–Wallis test followed by Dunn’s post hoc analysis or the Mann–Whitney *U* test. Categorical parameters were evaluated using Pearson’s Chi-square test or Fisher’s exact test. Hmisc (http://cran.r-project.org/web/packages/Hmisc) and corrplot (https://cran.r-project.org/package=corrplot) R packages were used to determine and visualise correlation significance between the analysed immune cells based on Spearman’s correlation coefficient. Immune-cell profiles were divided into two groups based on cut-off points according to the median (*≤* versus *>* median). The Cutoff Finder algorithm was additionally used to identify the optimal cut-off for each marker [33]. Overall survival analysis was performed by the Kaplan–Meier method and significance of differences between groups was assessed using the log-rank test. Univariate Cox proportional hazard regression analysis was performed on continuous measurements of cell markers using the survival (http://cran.r-project.org/web/packages/survival) R package. All reported *P* values were two-sided and *P* < 0.05 was considered statistically significant.

## Results

### Clinicopathological characteristics of the IPN and BilIN study cohort reveal significant differences

High-grade IPN (*n* = 65), including 54 IPNB, 11 ITPN, associated invasive BTC (*n* = 46) and non-tumorous biliary tissue (*n* = 64), all corresponding intraindividually were analysed as triplet samples, thereby immunohistochemically quantifying microenvironment immune cells (Fig. 1a, b). High-grade BilIN (*n* = 74), their associated invasive BTC (*n* = 74) and non-tumorous biliary tissue (*n* = 66) from the same patients were similarly selected for comparison [26]. Interestingly, overall survival analysis showed that patients with IPN- and BilIN-associated BTC had 5-year survival rates of 69.7% and 28.8%, respectively, thereby indicating that IPN-associated carcinogenesis is characterised by significantly better patient outcome (Fig. 1c). Next, we compared the clinicopathological characteristics of the IPN and BilIN groups (Table 1). This revealed that patients with IPN were younger than patients with BilIN (*P* = 0.042), but no significant differences in sex, distant metastasis (M) category or invasive tumour grading (G) were identified. The majority of IPN lesions had a distal localisation (43.1%, *n* = 28), while most BilIN lesions originated in the gallbladder (43.2%, *n* = 32, Table 1). Consistent with previous studies, a significant difference in distribution between intrahepatic, perihilar and distal CCA was also observed (*P* = 0.013). Significantly more patients with IPN were diagnosed with early-stage tumours (UICC 0–1), whereas most patients with BilIN had advanced tumour stages (UICC 3–4, *P* < 0.001). In addition, lymph node involvement was significantly higher in patients with BilIN (*P* < 0.001, Table 1). A comparison of the clinicopathological features of the two IPN subgroups revealed that the median age at diagnosis for patients with IPNB-associated carcinogenesis was higher than that of ITPN (*P* = 0.011, Supplementary Table S1). The anatomical localisation of IPN varied significantly within the cohort, as 20.4% (*n* = 11) of IPNB but 81.8% (*n* = 9) of ITPN were of intrahepatic biliary duct origin (*P* < 0.001), while no significant differences related to sex, histological subtype, UICC stage, TNM status or histological grade of the associated invasive BTC were observed between IPNB and ITPN (Supplementary Table S1).

### Alterations in the immune-cell composition of the microenvironment during IPN-associated carcinogenesis

**Fig. 2:**
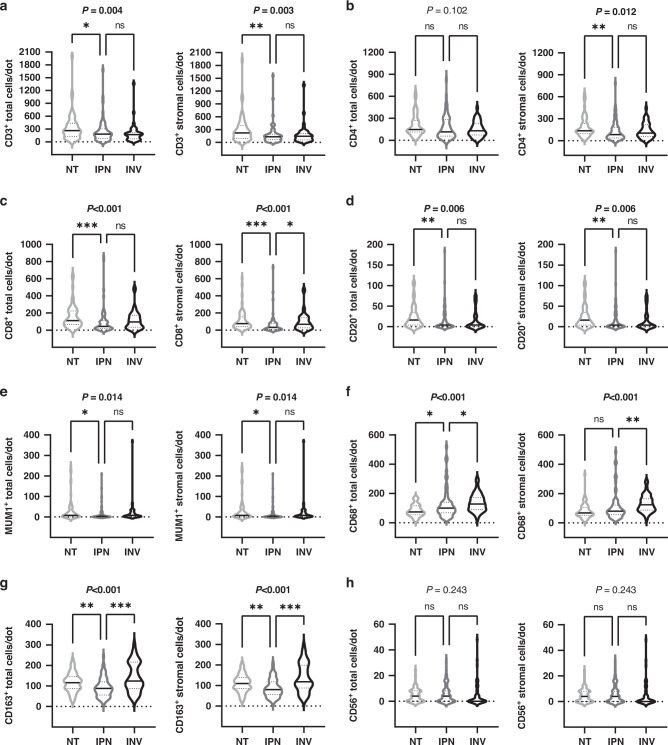
Distribution of total and stromal inflammatory cell infiltrates in intraductal papillary neoplasms of the biliary tract (IPN), their associated invasive BTC (INV) and non-tumorous tissue (NT). **a**–**h** Total and stromal counts of **a** CD3^+^ T lymphocytes (NT: *n* = 56; IPN: *n* = 65; INV: *n* = 41)**, b** CD4^+^ T lymphocytes (NT: *n* = 57; IPN: *n* = 65; INV: *n* = 41), **c** CD8^+^ T lymphocytes (NT: *n* = 57; IPN: *n* = 65; INV: *n* = 41), **d** CD20^+^ B lymphocytes (NT: *n* = 60; IPN: *n* = 65; INV: *n* = 43), **e** MUM1^+^ plasma cells (NT: *n* = 59; IPN: *n* = 65; INV: *n* = 42), **f** CD68^+^ macrophages (NT: *n* = 58; IPN: *n* = 65; INV: *n* = 42), **g** CD163^+^ macrophages (NT: *n* = 56; IPN: *n* = 65; INV: *n* = 42) and **h** CD56^+^ NK cells (NT: *n* = 57; IPN: *n* = 65; INV: *n* = 44). Significant *P* values of Kruskal–Wallis test are shown in bold, followed by Dunn’s post hoc analysis. *P < 0.05, **P < 0.01, ***P < 0.001; ns not significant, NA not applicable.

**Fig. 3:**
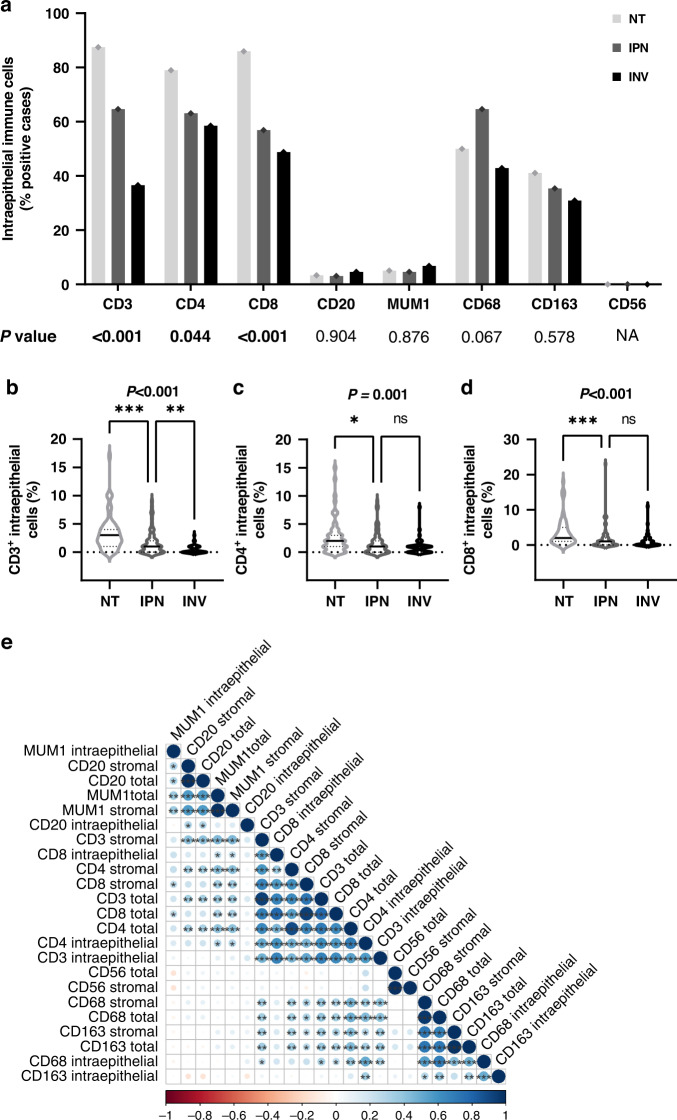
Distribution of intraepithelial inflammatory cell infiltrates in intraductal papillary neoplasms of the biliary tract (IPN), their associated invasive BTC (INV) and non-tumorous tissue (NT). **a** Percentage of cases showing any positive intraepithelial inflammatory cells in NT, IPN and INV. Significant *P* values of Pearson’s chi-squared test are shown in bold. **b**–**d** Proportion per 100 biliary epithelial cells of intraepithelial, **b** CD3^+^ T lymphocytes, **c** CD4^+^ T lymphocytes, **d** CD8^+^ T lymphocytes. Significant *P* values of Kruskal–Wallis test are shown in bold, followed by Dunn’s post hoc analysis. **e** Spearman’s correlation between all assessed inflammatory parameters in intraductal papillary lesions (IPN). Significant correlations were marked with asterisks: *P < 0.05; **P < 0.01; ***P < 0.001; ns not significant, NA not applicable.

We performed immunohistochemical analyses to determine the cellular composition and spatial organization of the IPN immune microenvironment during biliary carcinogenesis No statistically significant differences were observed between the immune-cell profiles of IPNB and ITPN in our cohort, thus justifying their combination throughout the study (Supplementary Fig. S2). CD3^+^ T lymphocytes predominated the total and stromal immune-cell infiltrate of IPN, with median cells per TMA dot of 180 and 132, respectively (Fig. 2a). Similarly, high CD4^+^ cell densities were observed in IPN lesions with a median of 116 total and 84 stromal cells per TMA dot (Fig. 2b). Interestingly, CD8^+^ T-cell counts were relatively low, accounting for a median CD8^+^ to CD4^+^ ratio of 0.38 in both total and stromal immune-cell compositions (Fig. 2b, c). We further compared the distribution of immune cells in IPN to that of their associated non-tumorous biliary tissue (NT) and invasive tissue lesions (INV). Stromal CD3^+^, CD4^+^ and CD8^+^ T-cell counts were significantly lower in IPN compared to the corresponding non-tumorous tissue (*P* = 0.002, *P* = 0.007 and *P* < 0.001, respectively), with CD8^+^ T-cell density diminishing by a factor of 2.4 (Fig. 2a–c). However, CD8^+^ T lymphocytes significantly increased in the stromal compartment as IPN transitioned to invasive carcinoma (*P* = 0.017, Fig. 2c), a finding that was not observed for CD3^+^ and CD4^+^ T cells in this patient cohort. CD20^+^ B lymphocytes and MUM1^+^ plasma cells were sparse, with a median of four cells per TMA dot each (Fig. 2d, e). Total and stromal CD20^+^ B lymphocytes and MUM1^+^ plasma cells significantly decreased between NT and IPN (*P* = 0.008 and *P* = 0.011, respectively) but not upon transition to invasive BTC (Fig. 2d, e). High levels of CD68^+^ and CD163^+^ macrophages were identified in IPN, with a median of 100 total and 80 stromal CD68^+^ cells per TMA dot and the ratio of CD163^+^ to CD68^+^ was 0.88 in the total and 1 in the stromal compartment (Fig. 2f, g). Macrophages showed unique distribution patterns throughout IPN-carcinogenesis. Total and stromal CD68^+^ macrophages displayed a stepwise increase from normal tissue through IPN, achieving a maximum in invasive BTC (*P* < 0.001, Fig. 2f). In contrast, total and stromal CD163^+^ macrophages initially declined in IPN but significantly increased in invasive BTC (*P* < 0.001). Consistently, the median CD163^+^ to CD68^+^ ratio increased from 0.88 in IPN to 0.99 in BTC (Fig. 2g). Throughout cholangiocarcinogenesis, CD56^+^ NK cell counts were low (median 4 cells per TMA dot), and no differences were observed at the transition from non-tumorous via IPN to invasive BTC (*P* = 0.243, Fig. 2h). As the short-distance interaction of immune cells with the tumour epithelium may differ from the stromal immune-cell profiles, we analysed the intraepithelial cell distribution of immune cells. Overall, more than half of the analysed IPN tissue samples exhibited intraepithelial CD3^+^ (64.6%, *n* = 42/65), CD4^+^ (63.1%, *n* = 41/65) and CD8^+^ (56.9%, *n* *=* 37/65) T-cell infiltration and the number of cases with intraepithelial CD3^+^, CD4^+^ and CD8^+^ cells gradually decreased throughout IPN-driven carcinogenesis (Fig. 3a). Interestingly, the proportion of intraepithelial CD3^+^, CD4^+^ and CD8^+^ T-cell infiltration decreased progressively from non-tumorous biliary tissue, through IPN, to invasive BTC, most significantly in early carcinogenesis (*P* < 0.001, *P* = 0.024 and *P* < 0.001, respectively; Fig. 3b–d). Further, CD20^+^ B lymphocytes and MUM1^+^ plasma cells were rarely found in the intraepithelial compartment of IPN cases (3.1%, *n* = 2/65 and 4.6%, *n* = 3/65, respectively), intraepithelial CD68^+^ macrophages were identified in over 64% (*n* = 42/65) of the analysed IPN tissue and no intraepithelial CD56^+^ NK cell infiltration was observed (Fig. 3a). In addition, no significant differences were found in the intraepithelial infiltration of CD20^+^, MUM1^+^, CD68^+^ and CD163^+^ immune cells (Supplementary Fig. S3). To validate these findings, immune-cell quantification was performed on whole-slide sections of arbitrarily selected cases which confirmed the results obtained by tissue microarray (Supplementary Fig. S1). Thus, intraepithelial T-cell infiltration significantly decreased in IPN-associated transition from non-tumorous epithelium via precursor lesion to invasive BTC, whereas B cells, plasma cells and macrophages retained unaltered profiles.

### Correlation of immune-cell distribution profiles in IPN-related cholangiocarcinogenesis

Intrahepatic, perihilar and distal CCA are not only defined by the WHO according to their anatomical localisation but represent molecularly distinct subgroups. Therefore, we studied the association between IPN immune profiles in a subgroup-specific manner, i.e., according to their anatomical localisation. Interestingly, merely minor differences between the three anatomical subgroups were observed. Only distal IPN were significantly associated with lower total CD68^+^ macrophage infiltration (*P* = 0.028; Supplementary Fig. S4A). Next, we compared the different UICC stages of the corresponding invasive BTC tissues and observed no significant alterations except for a trend towards higher CD163^+^ macrophage density advanced stage carcinoma (UICC 3–4) compared to the precancerous stage (UICC 0; *P* = 0.038; Supplementary Fig. S4B). Furthermore, correlation analysis of the total, stromal and intraepithelial immune-cell profiles (Fig. 3e) were performed. CD3^+^, CD4^+^ and CD8^+^ T lymphocytes showed strong, positive correlations in their total, stromal and intraepithelial distributions, both in intra- and intergroup comparisons (*r* = 0.54-0.92). Similar results were also observed for total and stromal CD20^+^ B lymphocytes and MUM1^+^ plasma cells (*r* = 0.57), as well as CD68^+^ and CD163^+^ macrophages (*r* = 0.68–0.98). In addition, stromal T lymphocytes correlated positively, but to a lesser extent, with stromal CD20^+^ B lymphocytes and MUM1^+^ plasma cells (*r* = 0.33–0.44), suggesting similar patterns of density variations for the components of the adaptive immunity. The proportions of intraepithelial B lymphocytes, plasma cells and total NK cells seemed to be independent of the remaining inflammatory components, most probably due to their low numbers (Fig. 3e). We further compared immune-cell distributions in invasive tissues of IPN-driven cholangiocarcinogenesis. In all three subtypes - intrahepatic, perihilar and distal CCA - the tumour microenvironment exhibited no statistically significant association with anatomical tumour localisation (Supplementary Fig. S5).

### Temporal heterogeneity of immune-cell distribution in biliary intraepithelial neoplasia compared to IPN

**Fig. 4:**
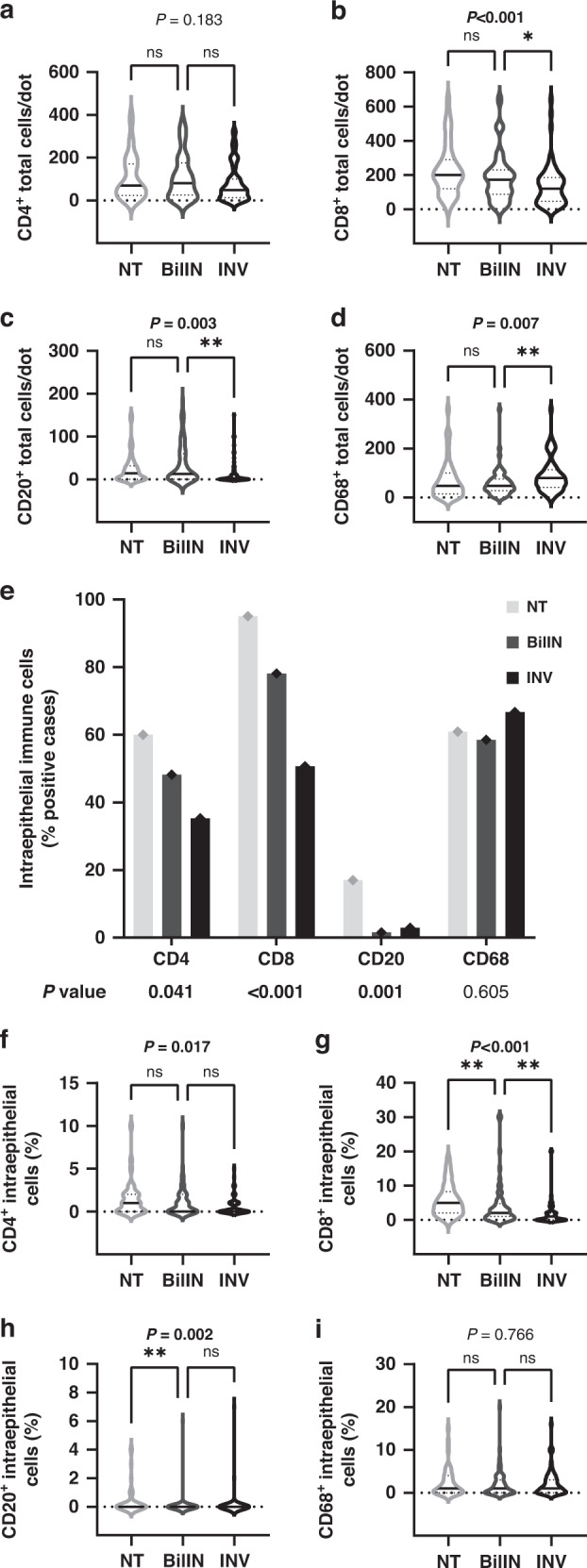
Distribution of inflammatory cell infiltrates in biliary intraepithelial neoplasia (BilIN), their associated invasive BTC (INV) and non-tumorous tissue (NT). **a**–**d** Total number of **a** CD4^+^ T lymphocytes (NT: *n* = 38; BilIN: *n* = 56; INV: *n* = 68)**, b** CD8^+^ T lymphocytes (NT: *n* = 42; BilIN: *n* = 65; INV: *n* = 66)**, c** CD20^+^ B lymphocytes (NT: *n* = 46; BilIN: *n* = 65; INV: *n* = 69) and **d** CD68^+^ macrophages (NT: *n* = 48; BilIN: *n* = 66; INV: *n* = 69). **e** Percentage of cases showing intraepithelial inflammatory cells in NT, IPN and INV. **f**–**i** Proportion per 100 biliary epithelial cells of intraepithelial, **f** CD4^+^ T lymphocytes, **g** CD8^+^ T lymphocytes, **h** CD20^+^ B lymphocytes and **i** CD68^+^ macrophages. Significant *P* values of Kruskal–Wallis (**a**–**d**, **f**–**i**) followed by Dunn’s post hoc analysis and Pearson’s chi-squared test (**e**) are shown in bold. *P < 0.05, **P < 0.01, ***P < 0.001; ns not significant, NA not applicable.

We hypothesised that IPN- and BilIN-driven tumour microenvironments could be immunologically distinct from each other. Total tissue-infiltrating T lymphocytes showed divergent distribution profiles in BilIN compared to IPN with a reversed median CD8^+^ to CD4^+^ T-cell ratio of 2.15 (Fig. 4a, b). This phenotype was caused by higher CD8^+^ T lymphocytes infiltration in BilIN (median 172 cells per TMA dot) compared to IPN (median 44 cells per TMA dot) and to the corresponding invasive carcinomas of BilIN cases (median 120 cells per TMA dot; Figs. 2c and  4b). Total CD8^+^ T-cell infiltration was not significantly decreased upon transition from non-tumorous epithelium to BilIN, as this change was only observed at the invasive BTC state (*P* = 0.043, Fig. 4b), whereas IPN displayed a significantly reduced total CD8^+^ infiltrate compared to non-tumorous epithelium (*P* < 0.001, Fig. 2c). Further, total CD20^+^ B lymphocytes significantly decreased in invasive BTC compared to BilIN (*P* = 0.002, Fig. 4c). Conversely, a significant increase in CD68^+^ macrophages was observed in invasive tumours compared to BilIN (*P* = 0.007, Fig. 4d). These findings are especially accentuated in extrahepatic cholangiocarcinogenesis (Supplementary Fig. S6) and seem to be independent of tumour UICC stage, as shown the same overall results were obtained for IPN and BilIN of BTC with UICC 2 (Supplementary Fig. S7).

Next, we compared intraepithelial immune-cell infiltrates during BilIN-associated carcinogenesis to that of IPN. Intraepithelial CD4^+^ and CD8^+^ T lymphocytes decreased from non-tumorous biliary tissue through BilIN to invasive BTC (*P* = 0.017, *P* < 0.001, respectively). However, this stepwise decrease of CD4^+^ and CD8^+^ infiltrates in BilIN-associated carcinogenesis was not mirrored in IPN-associated cholangiocarcinogenesis, in which the decrease of CD4^+^ and CD8^+^ T cells occurred mainly in IPN and was not observed in the transition to invasive BTC (Figs. 3d and  4e–g). A small and gradual increase in CD20^+^ B lymphocytes (*P* = 0.002) but not for CD68^+^ macrophages (*P* = 0.766) were noted in BilIN-driven carcinogenesis (Fig. 4h, i). Overall, we observed that intraepithelial CD8^+^ T cells exhibit a strong reduction only in IPN and not in the progression step to IPN-derived invasive BTC, whereas BilIN-associated cholangiocarcinogenesis showed a gradual decrease towards the transition to invasive BTC.

**Fig. 5:**
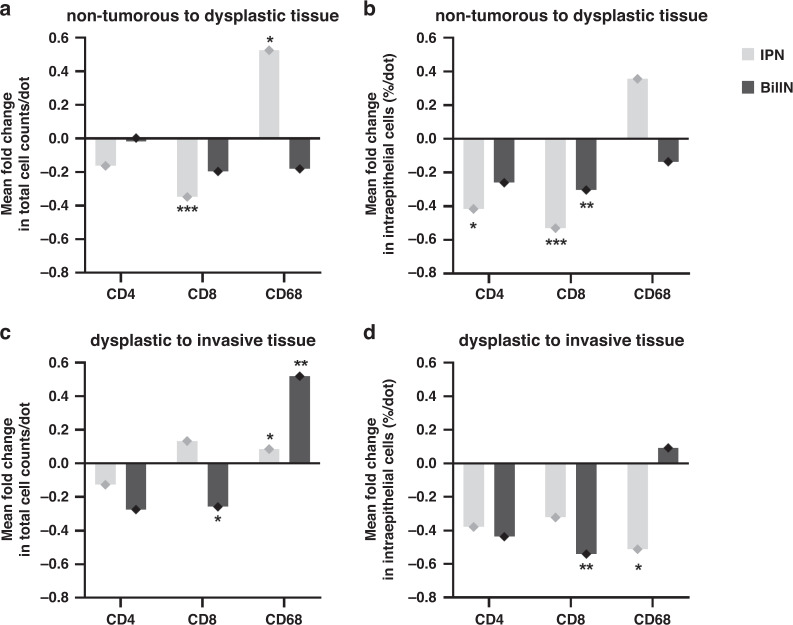
Changes in immune-cell distribution throughout IPN- and BilIN-associated carcinogenesis. **a**–**d** Each bar represents the mean fold change of total cell counts or intraepithelial cell percentages between (**a, b**) non-tumorous to dysplastic tissue and (**c**, **d**) between dysplastic and invasive tissue. Asterisks above bars indicate significance for comparisons shown in Figs. 2, 3 and 4. *P < 0.05, **P < 0.01, ***P < 0.001.

To further visualise the temporal distribution of total and intraepithelial immune-cell densities during biliary carcinogenesis, we plotted the mean fold change in cell densities for commonly evaluated immune cell markers (Fig. 5). Interestingly, the most significant changes in IPN-associated carcinogenesis were seen after the transition from the non-tumorous to the preinvasive IPN stage. Overall, a 0.51-fold increase in total CD68^+^ counts (*P* = 0.02), a 0.35-fold decrease in total CD8^+^ counts (*P* < 0.001) and a 0.53-fold decrease in intraepithelial CD8^+^ percentages (*P* < 0.001) were observed (Fig. 5a, b). In contrast, BilIN-associated carcinogenesis was characterised by significant changes in immune-cell composition at a later stage, when BilIN transitioned to invasive BTC. Here, we noted a 0.52-fold increase in total CD68^+^ counts (*P* = 0.007), a 0.26-fold decrease in total CD8^+^ counts (*P* = 0.043) and a 0.54-fold decrease in intraepithelial CD8^+^ percentages (*P* = 0.004, Fig. 5c, d). In conclusion, we revealed that IPN- and BilIN-tumour microenvironments undergo distinctive cellular changes at different steps throughout biliary carcinogenesis.

Regarding the impact of immune infiltrate composition on patient survival, we performed survival analysis to assess the prognostic value of the here profiled immune cells in IPN and BilIN on patient outcome. Cases were homogeneously divided into groups of low and high presence of individual immune-cell markers based on the median value (Supplementary Fig. S8). For all analysed markers, there was no significant association with patient overall survival even after optimal cut-off determination using Cutoff Finder [33]. Similarly, univariate Cox regression analysis showed no significant association between continuous measurements of immune-cell markers and overall survival (Supplementary Table S2). Cumulatively, our results indicate that the microenvironment of preinvasive BTC lesions is subject to complex and heterogenous spatiotemporal dynamics that cannot be captured by univariate survival analyses.

## Discussion

In this study, we demonstrated that the tumour microenvironment (TME) dynamically evolves during biliary carcinogenesis, thereby reflecting noteworthy changes in the distribution of tumour-infiltrating immune cells. By evaluating total, stromal, and intraepithelial tumour-infiltrating CD3^+^, CD4^+^ and CD8^+^ T lymphocytes, CD20^+^ B lymphocytes, MUM1^+^ plasma cells, CD68^+^ macrophages, CD163^+^ M2-like macrophages and CD56^+^ NK cells in non-tumorous biliary tissue specimens, corresponding precursor lesions and invasive carcinoma samples of the same patients, we deciphered the distribution and spatiotemporal evolution of tumour-infiltrating immune cells during biliary carcinogenesis. In addition, we focused on the comparison between IPN- and BilIN-mediated cholangiocarcinogenesis. Our findings revealed that: (1) total cell counts of CD8^+^ T and CD20^+^ B lymphocytes were strongly and significantly diminished in IPN. Further, the decrease in intraepithelial lymphocytic infiltration was more pronounced in IPN compared to BilIN. (2) In contrast to T lymphocytes, CD68^+^ and CD163^+^ macrophage infiltration increased in the tumour stroma and not tumour epithelium as biliary dysplastic tissue progressed to the invasive state. Thus, IPN- and BilIN-tumour microenvironments undergo temporally heterogeneous changes in immune-cell distribution.

A comprehensive understanding of the complex immune micromilieu is crucial for shaping future therapeutic perspectives of immune therapies in highly stromal tumours such as BTC, where chronic inflammation plays an etiopathogenetic role. Thus, redirecting the focus of immune profiling to the understudied precursor lesions is growingly encouraged. This trend is increasingly observed in several malignancies, including colorectal, oesophageal, and oral cancer [34]. However, attempts to characterise the crosstalk between neoplastic cells and the immune microenvironment in BTC are hitherto limited and basic knowledge of the composition of tumour-infiltrating immune cells in biliary precursor lesions is lacking. Here, we observed higher immune-cell densities in non-tumorous tissue of IPN-related BTC compared to the precursor IPN. On one hand, the persistent inflammatory state that accompanies biliary carcinogenesis could account for higher immune-cell counts in the non-tumorous biliary tissue of BTC patients [8, 9]. On the other hand, a relatively low immune-cell infiltration of high-grade IPN lesions may reflect the ability of dysplastic cells to evade immunosurveillance [35, 36].

Immune evasion and exclusion are thought to be mediated by various oncogenic pathways. One suggested mechanism involves hyperactivity of the WNT/β-catenin signalling pathway, which has been shown to be of importance in IPN-driven tumorigenesis [20–22]. Consequently, neoplastic cells reduce the expression of chemokines such as CCL4 and CCL5, thereby leading to a diminished antigen-specific CD8^+^ T-cell priming [37, 38]. CD8^+^ T cells are powerful contributors to antitumour immunity, most importantly in the priming phase, as they detect tumour antigens presented by MHCI molecules. Once activated, they exert their effector potential by targeting cancer cells via exocytosis of cytotoxic granules and induction of FasL-mediated apoptosis [39, 40]. Thus, limiting CD8^+^ T-cell infiltration provides a survival benefit to neoplastic tissue. Another factor that could potentially explain the relatively low immune-cell densities in IPN is the successful establishment of a physical barrier to immune cell penetration by the preinvasive IPN cells. The tumour-reactive stroma is a pivotal element of carcinogenesis and tumour progression, especially in highly desmoplastic tumours such as BTC. Stromal cells, particularly cancer-associated fibroblasts (CAFs), are potent effectors of carcinogenesis, starting at preinvasive stages, as seen in prostate and breast cancer [41–43]. Importantly, CAFs are involved in limiting intratumoural cytotoxic T-lymphocyte infiltration through various mechanisms, including extracellular matrix production and CXCL12 secretion [44, 45]. CAFs are increasingly seen as an immunologic barrier against CD8^+^ T-cell-mediated antitumour effects, supported by a negative correlation of CD8^+^ T-cell infiltration and CAF density in tumours [46–48]. In line with our findings on CD8^+^ cell dynamics in BTC, this could suggest that CAFs are potentially recruited earlier during IPN-associated tumorigenesis compared to its BilIN counterpart. In addition, the papillary growth pattern of IPN could be of particular interest in the setting of immune evasion. The protrusion of IPN lesions into the bile duct lumen could possibly reduce immune-cell infiltration, thereby minimising direct contact between neoplastic and immune cells. As papillary and tubular intraluminal structures such as IPN have a partial connection to the wall of the affected biliary ducts, this physical barrier may serve as a bottleneck for invading lymphocytes. Taken together, these aspects provide a possible explanation to the TME-composition differences between papillary lesions such as IPN and flat precursor lesions such as BilIN. The here observed significant difference in terms of overall survival between our IPN and BilIN cohorts may also be justified by the morphological nature of IPN lesions. The intraductal growth and papillary structure of IPN may render an earlier development of obstructive symptoms, detectable changes on imaging studies, and therefore higher chances for a prompt management.

Studies aiming at investigating the dynamics of tissue microenvironment in precursor lesions of gastrointestinal and pancreatobiliary tumours are limited. Since IPN-driven tumorigenesis is regarded as the biliary equivalent to the colorectal adenoma–carcinoma sequence [4], comparing both entities might offer new insights into the interplay between immune and neoplastic cells. Interestingly, higher densities of CD68^+^ macrophages and CD4^+^ T lymphocytes with median CD8 to CD4 ratios between 0.3 and 0.4 were reported in polypoid premalignant colorectal lesions compared to nonpolypoid lesions [49]. These results are in line with the here observed differences between IPN and BilIN, two precursor lesions with distinct histomorphological features. Further, the authors noted a lower CD8 to CD4 ratio in nonpolypoid compared to polypoid lesions [49], as opposed to the here observed prevalence of CD8^+^ cells in BilIN. On one hand, we found that total CD8^+^ cells significantly decreased in BilIN-associated BTC, which could represent a possible feature of BilIN-driven carcinogenesis. On the other hand, we showed that stromal CD8^+^ cell density increased in IPN-associated BTC tissue, a trend that might characterise IPN-tumorigenesis. These data, therefore, suggest that CD8^+^ lymphocytes may be subject to different dynamics during the preinvasive-to-malignant transition, depending on the histomorphology of the precursor lesion.

Furthermore, IPN of the biliary tract are viewed as morphologically and clinicopathologically comparable to their pancreatic counterpart: the intraductal papillary mucinous neoplasm (IPMN) [50]. A recent study showed that stromal CD8^+^ T cells gradually decreased during progression from IPMN to invasive pancreatic cancer [51], as opposed to the trend we observed in IPN. This difference might assert the putative role of the WNT/β-catenin pathway in IPN-carcinogenesis, as mutations in APC and CTNNB1 are not or are rarely found in IPMN [21, 52, 53]. The hyperactivation of the β-catenin signalling pathway has been linked to elevated expressions of CTLA-4 and IL-10 in human melanomas, thereby establishing an immunosuppressive tumour microenvironment that favours tumour-cell proliferation [38, 54]. Interestingly, and consistent with our results, the authors also reported a progressive increase in CD68^+^ macrophages during IPMN carcinogenesis [51], thereby highlighting the role of TAMs in the natural history of both biliary and pancreatic cancer.

TAMs are increasingly gaining interest as central elements of the innate immune sensing of tumours. These cells constitute a heterogeneous, functionally plastic group, as they manifest proinflammatory, anti-tumorigenic (M1-phenotype) and anti-inflammatory, pro-tumorigenic (CD68^+^/CD163^+^ M2-phenotype) functions. High tissue infiltration of M2 TAMs has been associated with a more aggressive tumour behaviour, as they promote matrix remodelling, tumour-cell invasion, intravasation and angiogenesis [42, 55, 56]. High densities of CD163^+^ macrophages were correlated with the occurrence of extrahepatic metastases in cholangiocarcinoma, possibly through the activation of signalling pathways involved in epithelial-to-mesenchymal transition [57]. Here, we observed increased numbers of CD68^+^ macrophages in invasive tumours compared to high-grade IPN and BilIN. Interestingly, we also revealed an increase in the CD163 to CD68 ratio during the transition from IPN to invasive BTC, thereby highlighting the tumour-promoting role of M2 TAMs.

A common feature of both IPN- and BilIN-associated carcinogenesis is the here observed progressive decrease in intraepithelial T-lymphocyte infiltration. Consequently, biliary tissue gradually acquires a so-called “altered” immunophenotype [58, 59], an intermediate state between highly infiltrated, “hot” tumours and non-inflamed “cold” tumours. This progressive “cooling” throughout cholangiocarcinogenesis could account for the acquired intratumoral immune tolerance in advanced BTC. This evolutionary “exhaustion” of proinflammatory immune cells could justify the ongoing challenges faced by immuno-monotherapy regimens in advanced BTC. Recently, the interim results of the phase III TOPAZ-1 trial investigating gemcitabine, cisplatin and the PD-L1 inhibitor durvalumab were released, stating that first-line chemoimmunotherapy significantly improved overall survival in advanced BTC patients without exacerbating toxicity [60]. We here demonstrated that TME dynamics in BTC progressively lead to an immune exclusion rather than immune desertion, therefore supporting the putative benefits of immunotherapy in combination regimens.

High densities of intratumoral CD8^+^ cells have been associated with a better outcome of BTC patients [26, 61, 62], a finding that was could not be observed in our IPN- and BilIN cohort. Larger, well-defined, and immunologically well-characterised IPN and BilIN cohorts are still missing, so that conclusions on the influence of immune-cell composition on patient survival could not be affirmatively drawn. The complexity of immune-cell dynamics in BTC impedes the depiction of a well-defined tumour microenvironment landscape and prompts future investigations. An in-depth analysis of T-cell subtypes (i.e., Tregs, Th1, Th2, Th9 and Th17 cells), CD103^+^ dendritic cells and myeloid-derived suppressor cells might reveal pivotal changes in TME during biliary tumorigenesis and should be the aim of prospective studies.

In conclusion, our study represents the first attempt to exhaustively characterise the complex tumour immune microenvironment in well-defined precursor lesions of biliary tract cancer, thereby revealing major changes with regards to immune-cell composition during IPN- and BilIN progression at different stages of cholangiocarcinogenesis. These findings represent a fragment of the whole picture of a deeper understanding of antitumour immunity in biliary carcinogenesis and may serve as a solid basis for future studies focusing on immunomodulation in BTC.

## Supplementary information

Supplematary Material

Reproducibility checklist

## Data Availability

The authors confirmed that the data supporting the findings of this study are available within the article and/or supplementary materials.
