# Supplementary material for: Spatiotemporal analysis of tumour-infiltrating immune cells in biliary carcinogenesis
Source: Br J Cancer. 2022 Sep 6;127(9):1603–14. doi: 10.1038/s41416-022-01933-0 (PMC9596479; doi:10.1038/s41416-022-01933-0)
Supplement: Supplementary file 1 — Supplematary Material [file 41416_2022_1933_MOESM1_ESM.docx]

**Spatiotemporal analysis of tumour-infiltrating immune cells in biliary carcinogenesis**

Alphonse Charbel^1,2^, Luca Tavernar^1,2^, Thomas Albrecht^1,2^, Fritz Brinkmann^1,2^, Joanne Verheij^3^, Eva Roos^3^, Monika Nadja Vogel^4^, Bruno Köhler^2,5^, Christoph Springfeld^2,5^, Alexander Brobeil^1,6^, Peter Schirmacher^1,2^, Stephan Singer^7^, Arianeb Mehrabi^2,8^, Stephanie Roessler^1,2*^, Benjamin Goeppert^1,2,9*^

^1^ Institute of Pathology, University Hospital Heidelberg, Heidelberg, Germany

^2^ Liver Cancer Centre Heidelberg (LCCH), Heidelberg, Germany

^3^ Department of Pathology, Amsterdam UMC, University of Amsterdam, Amsterdam, The Netherlands

^4^ Diagnostic and Interventional Radiology, Thoraxklinik at University Hospital Heidelberg, Heidelberg, Germany

^5^ Department of Medical Oncology, National Centre for Tumour Diseases, University Hospital Heidelberg, Heidelberg, Germany

^6^ Tumor Bank Unit, Tissue Bank of the National Center for Tumor Diseases, Heidelberg, Germany.

^7^ Institute of Pathology, University of Tübingen, Tübingen, Germany.

^8^ Department of General, Visceral and Transplantation Surgery, University Hospital Heidelberg, Heidelberg, Germany

^9^ Institute of Pathology, Hospital RKH Kliniken Ludwigsburg, Ludwigsburg, Germany

^*^These authors contributed equally to this work

**Supplementary Tables**

**Supplementary Table S1:** Clinicopathological characteristics of patients with high-grade intraductal papillary (IPNB) or intraductal tubulopapillary (ITPN) neoplasms of the biliary tract

|  | **Total** | **IPNB** | **ITPN** | **p-value** |
| --- | --- | --- | --- | --- |
| **Parameter, N (%)** | 65 (100.0) | 54 (83.1) | 11 (16.9) |  |
| **Age** |  |  |  |  |
| Median, years [IQR*] | 64 [39-81] | 67.5 [39-81] | 54 [40-72] | **0.011**† |
| **Sex** |  |  |  |  |
| Male | 40 (61.5) | 35 (64.8) | 5 (45.5) |  |
| Female | 25 (38.5) | 19 (35.2) | 6 (54.5) | 0.311^‡^ |
| **Localisation** |  |  |  |  |
| Intrahepatic | 20 (30.8) | 11 (20.4) | 9 (81.8) |  |
| Perihilar | 17 (26.2) | 16 (29.6) | 1 (9.1) |  |
| Distal | 28 (43.1) | 27 (50.0) | 1 (9.1) | **<0.001**^‡^ |
| **Histology precursor** |  |  |  |  |
| Pancreatobiliary | 43 (66.2) | 33 (61.1) | 10 (90.9) |  |
| Gastric | 5 (7.7) | 5 (9.3) | 0 (0.0) |  |
| Intestinal | 15 (23.1) | 15 (27.8) | 0 (0.0) |  |
| Oncocytic | 2 (3.1) | 1 (1.9) | 1 (9.1) | 0.077^‡^ |
| **UICC^§^** |  |  |  |  |
| UICC 0 | 6 (9.2) | 6 (11.1) | 0 (0.0) |  |
| UICC 1 | 13 (20.0) | 11 (20.4) | 2 (18.2) |  |
| UICC 2 | 22 (33.8) | 20 (37.0) | 2 (18.2) |  |
| UICC 3 | 9 (13.8) | 7 (13.0) | 2 (18.2) |  |
| UICC 4 | 4 (6.2) | 2 (3.7) | 2 (18.2) |  |
| NA | 11 (16.9) | 8 (14.8) | 3 (27.3) | 0.275^‡^ |
| **pT** |  |  |  |  |
| Tis | 6 (9.2) | 6 (11.1) | 0 (0.0) |  |
| T1 | 21 (32.3) | 15 (27.8) | 6 (54.5) |  |
| T2 | 29 (44.6) | 25 (46.3) | 4 (36.4) |  |
| T3 | 7 (10.8) | 7 (13.0) | 0 (0.0) |  |
| T4 | 2 (3.1) | 1 (1.9) | 1 (9.1) | 0.169^‡^ |
| **pN** |  |  |  |  |
| N0 | 38 (58.5) | 33 (61.1) | 5 (45.5) |  |
| N1 | 13 (20.0) | 11 (20.4) | 2 (18.2) |  |
| N2 | 1 (1.5) | 1 (1.9) | 0 (0.0) |  |
| NA | 13 (20.0) | 9 (16.7) | 4 (36.4) | 0.497^‡^ |
| **M** |  |  |  |  |
| M0 | 61 (93.8) | 52 (96.3) | 9 (81.8) |  |
| M1 | 4 (6.2) | 2 (3.7) | 2 (18.2) | 0.130^‡^ |
| **G** |  |  |  |  |
| G1 | 2 (3.1) | 2 (3.7) | 0 (0.0) |  |
| G2 | 46 (70.8) | 36 (66.7) | 10 (90.9) |  |
| G3 | 11 (16.9) | 10 (18.5) | 1 (9.1) |  |
| NA (Tis) | 6 (9.2) | 6 (11.1) | 0 (0.0) | 0.412^‡^ |
| **Invasive component** |  |  |  |  |
| Yes | 59 (90.8) | 48 (88.9) | 11 (100.0) |  |
| No | 6 (9.2) | 6 (11.1) | 0 (0.0) | 0.579^‡^ |
|  |  |  |  |  |

* IQR, interquartile range; ^†^ Kruskal-Wallis test; ‡ χ² test; ^§^ 8th edition; cases with pNx had no lymph nodes resected, therefore, UICC status could not be assessed; bold values indicate statistical significance with p<0.05

**Supplementary Table S2**: Univariate Cox proportional hazards model for overall survival in patients with IPN or BilIN precursor lesions

| **Variable** | **HR** | **(95% CI)** | **p-value** |
| --- | --- | --- | --- |
| **IPN** |  |  |  |
| **Total counts** |  |  |  |
| CD3 | 1.00 | (0.998-1.002) | 0.92 |
| CD4 | 1.00 | (0.999-1.004) | 0.17 |
| CD8 | 1.00 | (0.998-1.003) | 0.88 |
| CD20 | 0.99 | (0.956-1.020) | 0.56 |
| MUM1 | 1.00 | (0.990-1.010) | 0.68 |
| CD68 | 1.00 | (0.992-1.003) | 0.41 |
| CD163 | 1.00 | (0.986-1.008) | 0.50 |
| CD56 | 1.00 | (0.952-1.101) | 0.63 |
| **Stromal counts** |  |  |  |
| CD3 | 1.00 | (0.997-1.002) | 0.88 |
| CD4 | 1.00 | (0.999-1.005) | 0.15 |
| CD8 | 1.00 | (0.997-1.004) | 0.69 |
| CD20 | 0.99 | (0.956-1.020) | 0.44 |
| MUM1 | 1.00 | (0.990-1.010) | 0.46 |
| CD68 | 1.00 | (0.991-1.003) | 0.32 |
| CD163 | 1.00 | (0.985-1.008) | 0.44 |
| CD56 | 1.00 | (0.952-1.101) | 0.63 |
| **BilIN** |  |  |  |
| **Total counts** |  |  |  |
| CD4 | 1.00 | (0.996-1.004) | 0.88 |
| CD8 | 1.00 | (0.997-1.002) | 0.77 |
| CD20 | 1.00 | (0.995-1.010) | 0.49 |
| CD68 | 1.00 | (0.993-1.004) | 0.64 |

*IPN:* intraductal papillary neoplasms
*BilIN:* biliary intraepithelial neoplasia

**Supplementary Figures**


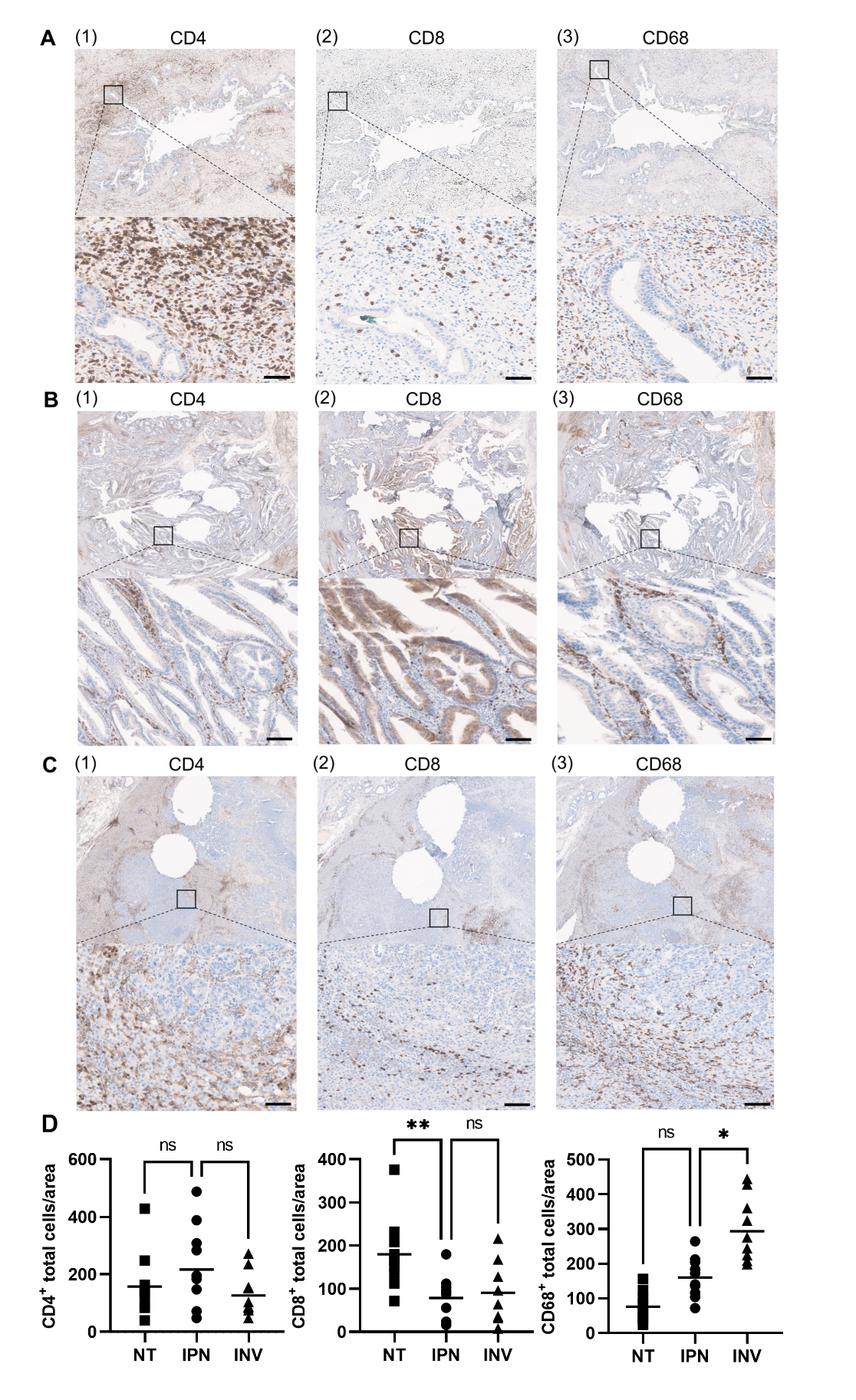


**Supplementary Figure S1. Representative images of IPN-related cases with whole slide immunohistochemical cell quantification. (A)** Non-tumorous tissue with CD4 (1), CD8 (2) and CD68 (3) antibody staining. Original magnification: 20x. **(B)** IPNB with CD4 (1), CD8 (2) and CD68 (3) antibody staining. Original magnification: 20x. **(C)** IPNB-driven BTC with CD4 (1), CD8 (2) and CD68 (3) antibody staining. Original magnification: 20x. The black scale bar represents 100μm for each panel. **(D)** Immune cell quantification in 10 randomly selected circular areas of 1 mm diameter on whole-slide sections.


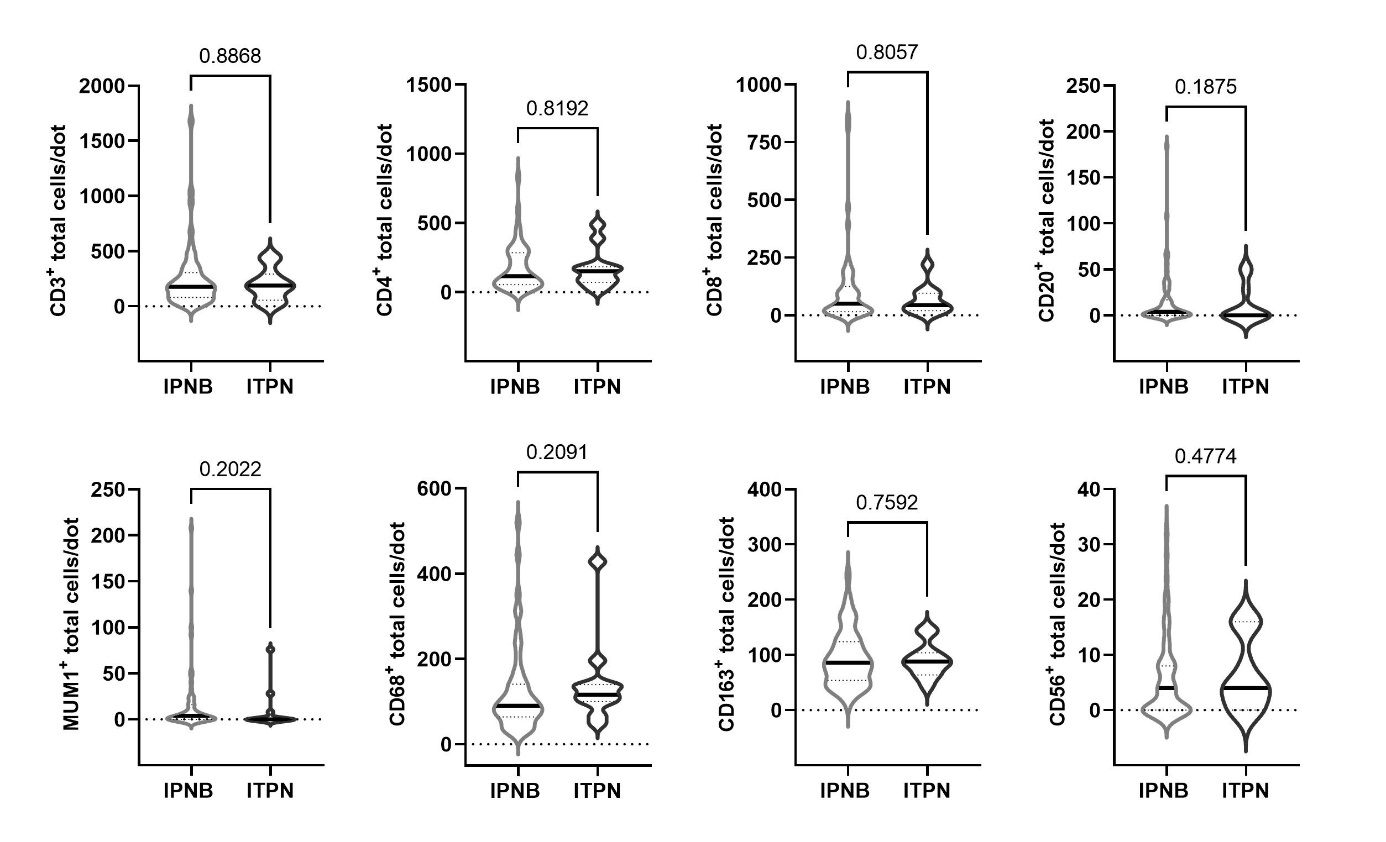


**Supplementary Figure S2. Distribution of intraepithelial inflammatory cell infiltrates in intraductal papillary neoplasms of the biliary duct (IPNB, *n*=54) and intraductal tubulopapillary neoplasm of the bile duct (ITPN, *n*=11).** *P*-values of the Mann-Whitney U test are shown.


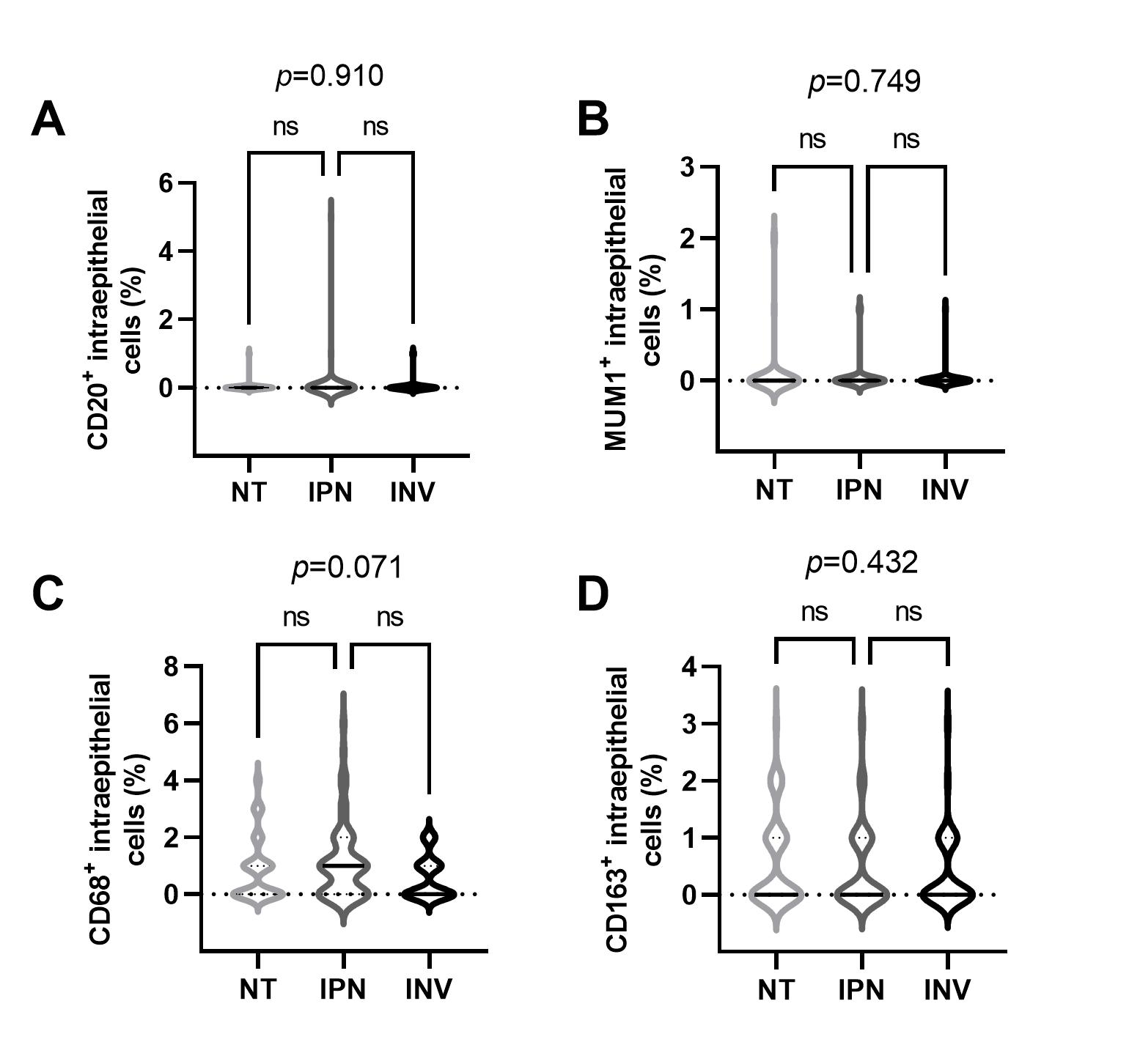


**Supplementary Figure S3. Distribution of intraepithelial inflammatory cell infiltrates in intraductal papillary neoplasms of the biliary tract (IPN), their associated invasive BTC (INV) and non-tumorous tissue (NT). (A-D)** Proportion per 100 biliary epithelial cells of intraepithelial (**A)** CD20^+^ B lymphocytes (**B)** MUM1^+^ plasma cells (**C)** CD68^+^ macrophages and (**D)** CD163^+^ macrophages. Significant p-values of Kruskal-Wallis test are shown in bold, followed by Dunn’s posthoc analysis. *p<0.05, **p<0.01, ***p<0.001; ns, not significant; NA, not applicable.

**
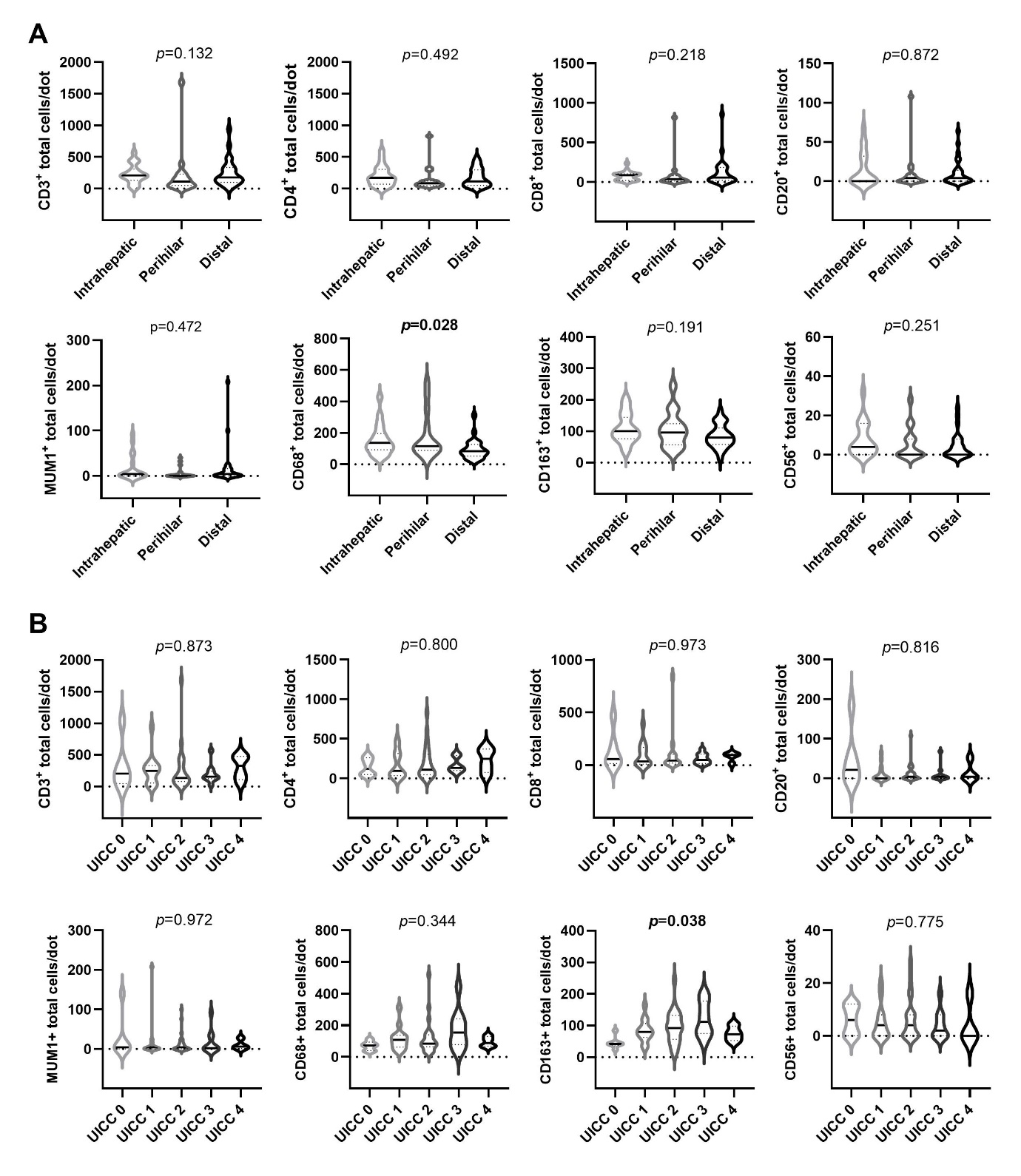
**

**Supplementary Figure S4. Distribution of inflammatory cell infiltrates in IPN according to (A) location and (B) UICC-stage of associated invasive biliary tract cancer.** Significant p-values of Kruskal-Wallis test are shown in bold, followed by Dunn’s post hoc analysis. *p<0.05.

**
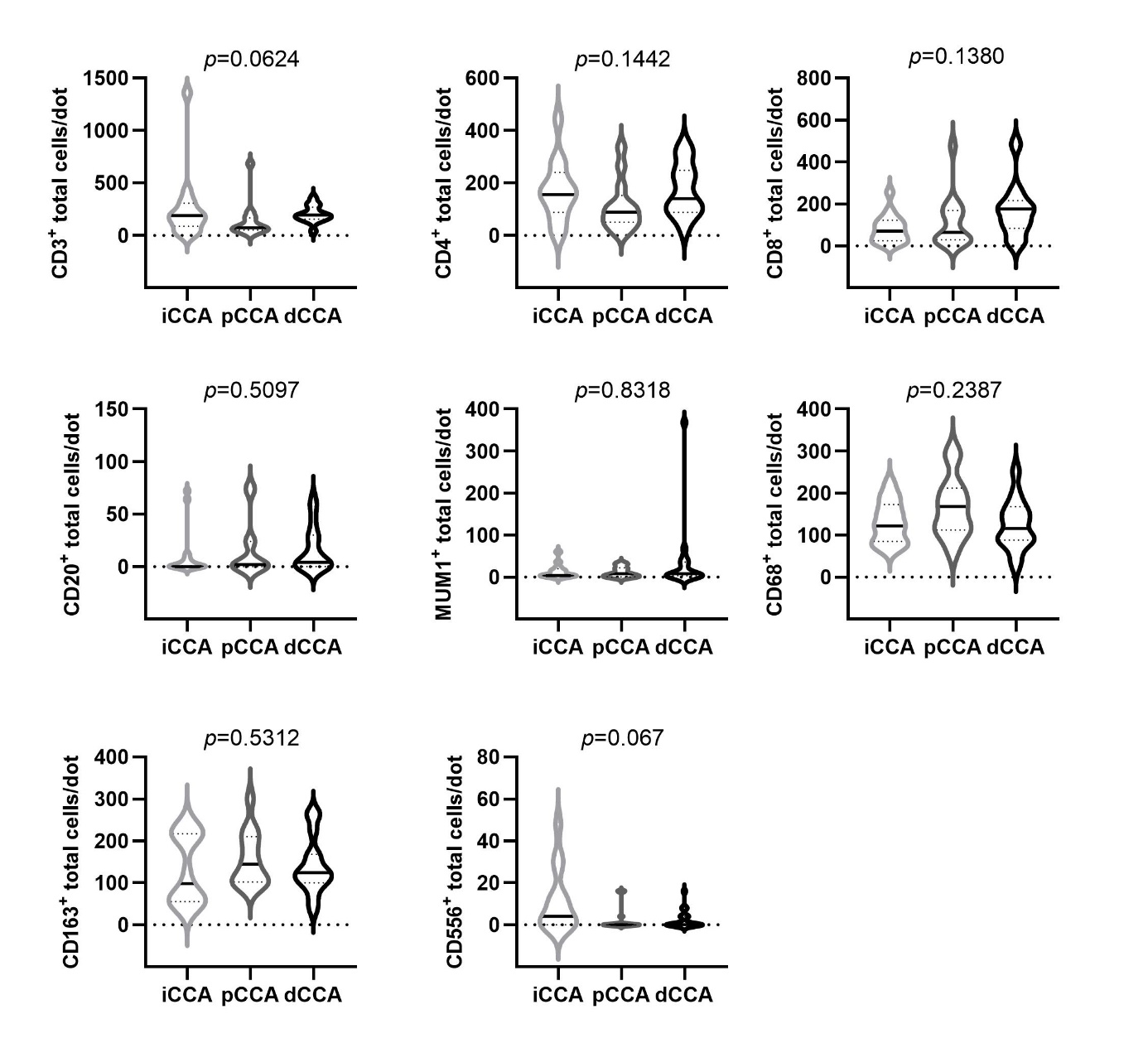
**

**Supplementary Figure S5. Distribution of inflammatory cell infiltrates in IPN-associated biliary tract cancer according to BTC-subtype.** *P*-values of Kruskal-Wallis test are shown.

**
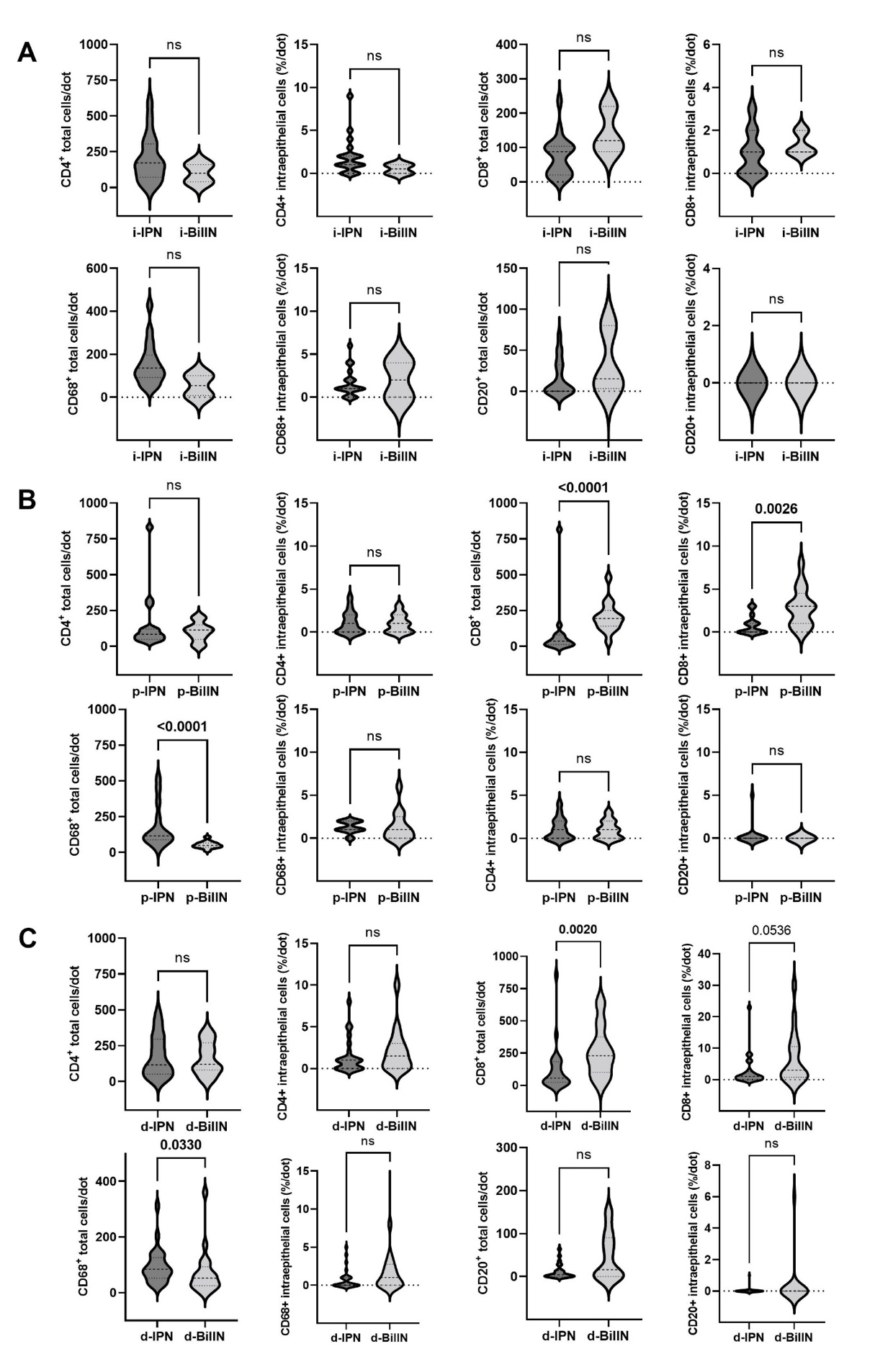
**

**Supplementary Figure S6. Distribution of inflammatory cell infiltrates in IPN and BilIN according to the localisation of the precursor lesions.** **(A)** Comparison of indicated immune cell infiltrates in intrahepatic (IPN n=20; BilIN n=3), **(B)** perihilar (IPN n=17; BilIN n=17) and **(C)** distal IPN or BilIN lesions (IPN n=28; BilIN n=22). P-values of the Mann-Whitney U test are shown; i = intrahepatic, p = perihilar; d = distal.

**
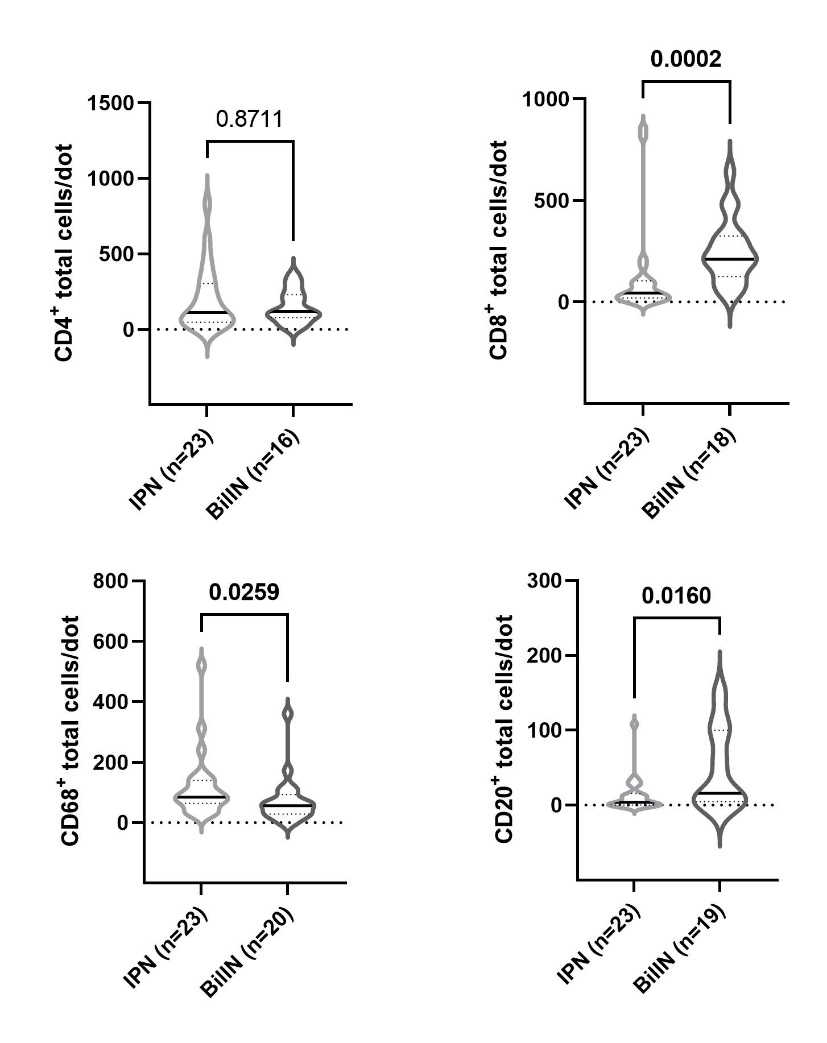
**

**Supplementary Figure S7. Distribution of inflammatory cell infiltrates in IPN and BilIN corresponding to UICC 2 BTC.** *P*-values of the Mann-Whitney U test are shown; significant *p*-values are shown in bold.

**
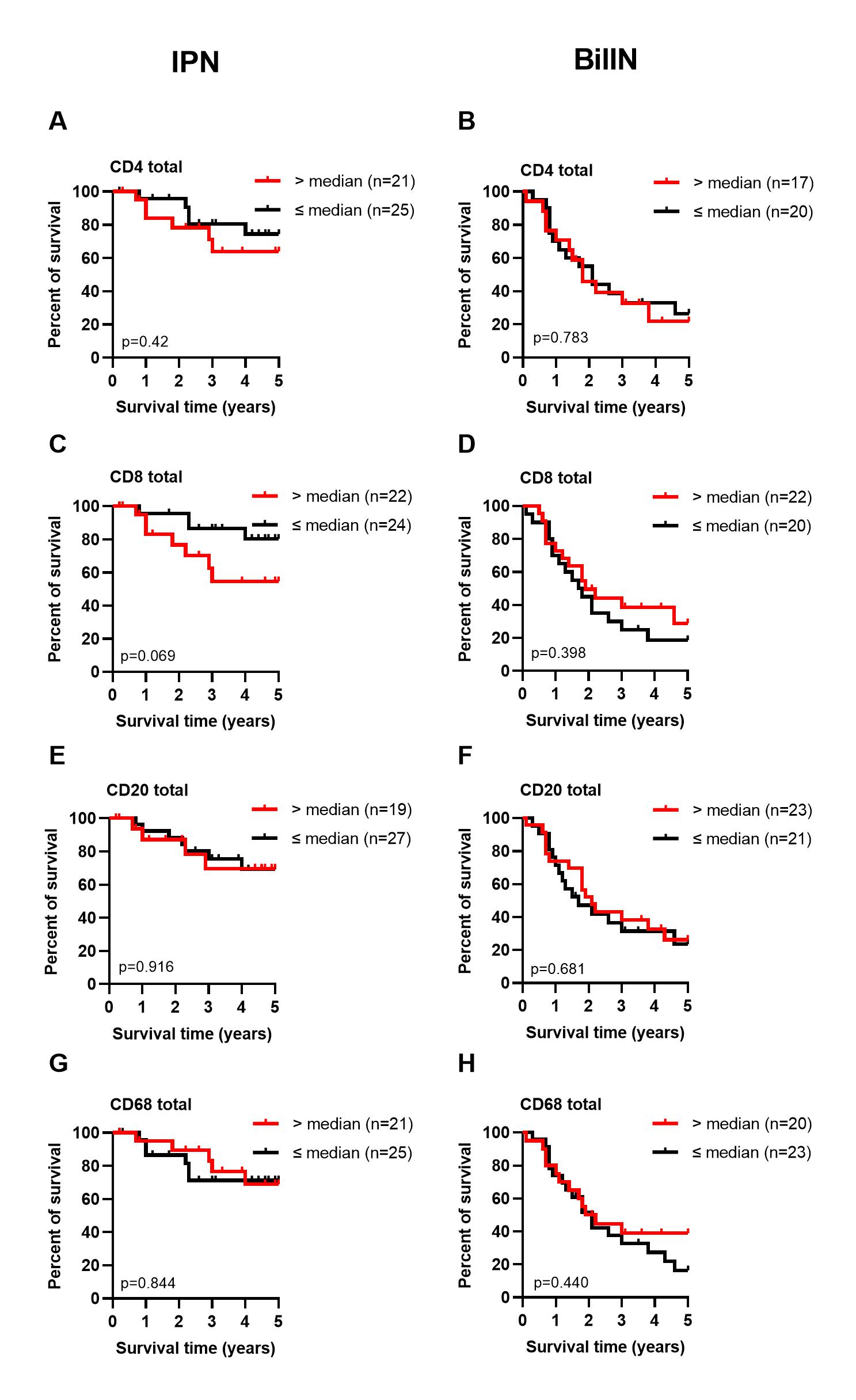

Supplementary Figure S8. Overall survival probability in patients with papillary precursor lesions of biliary tract cancer (A, C, E, G) and high-grade BilIN (B, D, F, H) stratified for inflammatory cell infiltrates**. Kaplan–Meier curves depict overall survival probability stratified for total immune cell infiltrate of **(A, B)** CD4^+^ T lymphocytes, **(C, D)** CD8^+^ T lymphocytes, **(E, F)** CD68^+^ macrophages and **(G, H)** CD20^+^ B lymphocytes**.** p-values calculated by log-rank test.
